# Cranberry constituents prevent SOS-mediated filamentation of uropathogenic *Escherichia coli*

**DOI:** 10.1128/iai.00600-24

**Published:** 2025-04-10

**Authors:** Tracy Prinster, Alistair Harrison, Christopher Dick, Dennis J. Horvath, Birong Li, Grace Sievers, Revanth Madamsetty, Jingwen Zhang, Kevin M. Mason, Christina Khoo, Sheryl S. Justice

**Affiliations:** 1 The Abigail Wexner Research Institute at Nationwide Children’s 51711, Columbus, Ohio, USA; 2 The Ohio State University College of Medicine 12305 , Columbus, Ohio, USA; 3 The College of Nursing, The Ohio State University 15953 , Columbus, Ohio, USA; 4 Ocean Spray Cranberries, Inc, Lakeville, Massachusetts, USA; University of California Davis, Davis, California, USA

**Keywords:** urinary tract infection, cranberry, *Escherichia coli*, filamentation

## Abstract

The diameter, length, and shape of bacteria are maintained with such high fidelity that these parameters are classically used as metrics in the distinction of bacterial species. Increasing evidence indicates that bacteria transiently shift their shapes into distinctive morphologies in response to environmental changes. Elongation of bacterial length into a filamentous shape provides unique survival advantages for many bacterial species. Analysis of 42 clinical isolates of uropathogenic *Escherichia coli* (UPEC) revealed that filamentation to host-derived antimicrobials is a conserved phenotype. Therefore, we hypothesize that filamentation represents a conserved mechanism of pathogenic bacterial persistence that can be targeted for narrow-spectrum, anti-virulence therapies. We demonstrate that cranberries prevent SulA-mediated filamentation of UPEC. Furthermore, we identify multiple fractions of cranberries that retain anti-filamentation properties. These studies provide mechanistic insight into the clinical efficacy of cranberry for patients with recurrent urinary tract infections. Inhibition of filamentation represents a novel approach to promote bacterial pathogen susceptibility to immune and antibiotic-mediated clearance to attenuate disease.

## INTRODUCTION

The introduction of antibiotics to treat infection was a pivotal event in the extension of the human lifespan. The limited number of new antimicrobials in the pipeline, combined with the significant rise in antibiotic-resistant pathogens, is threatening our ability to effectively combat bacterial infections (1–3). The Centers for Disease Control and the World Health Organization consider antimicrobial resistance a global threat to human health (4). In addition, evidence is mounting for the long-term effects of dysbiosis induced by broad-spectrum antibiotics on the human microbiome (5). Taken together, there is a critical need for new approaches to treat bacterial infections that target specific pathogenic lifestyles as well as reduce the potential for accumulation of resistant organisms and microbiome dysbiosis.

Antibiotic-resistant organisms arise from error-prone repair and horizontal gene transfer that is mediated by the inducible Save our Souls (SOS) response (6–12). The SOS regulon is a conserved bacterial stress response that includes multiple enzymes that repair DNA damage. In addition, the SOS regulon includes a cell division inhibitor, SulA, that specifically binds to the FtsZ monomer to prevent the formation of the septal ring (13–16). Continued growth during the SOS response results in the formation of filamentous morphotypes that are 10–50 times longer than the bacillary form and contain multiple copies of the genome (17, 18). The nature of the SOS response in DNA repair requires that the regulon members be tightly controlled to ensure transient activity only when DNA damage is present (19–22). In addition to transcriptional control to initiate and terminate the SOS regulon, SulA is rapidly degraded by the general cytoplasmic protease, Lon, to rapidly restore cell division (23–25).

Although the SOS response was initially described as a mechanism to repair DNA damage with high fidelity, this system is increasingly also recognized as being critical for bacterial pathogenesis, development of persister cells, and bacterial adaptation (6, 12, 26–29). Antimicrobials (e.g., antibiotics, reactive oxygen and nitrogen species, antimicrobial peptides) are known inducers of the SOS response, which can induce biofilm formation and antimicrobial tolerance (9, 12, 27, 30). Most recently, psychoactive medications have been shown to have off-target effects, stimulating the SOS response and horizontal gene transfer in bacteria (31–33). The important role of the SOS response in pathogenesis and antimicrobial resistance has led multiple groups to investigate SOS regulatory elements as new antimicrobial targets (6, 8, 27, 34, 35).

Bacterial filamentation is recognized as a survival strategy that enables bacteria to cope with various environmental stresses, including antimicrobial exposure, protist predation, and exposure to host phagocytes (17, 18, 36–38). Morphological plasticity is observed in multiple pathogens in response to a plethora of environmental and host stressors (18, 39, 40). The filamentous morphotype promotes resistance to killing by professional and predatory phagocytes as well as promotes tissue dissemination (38, 41–47). Antibiotic-resistant mutations are associated with morphological elongation even in the absence of antibiotics (48). The size and shape of target particles modulate immune function and inflammasome activation (49). Thus, differentiation to a filamentous morphology is now increasingly recognized as a generalized pathogenic lifestyle in multiple genera of bacteria that cause diseases and also represents a major hurdle in the treatment of bacterial infections (18, 50).

Bacterial filamentation during infection was first observed in the urine of patients with symptomatic urinary tract infections (UTIs) and in preclinical models of UTI (51–54). Among the many bacterial species known to cause UTIs, uropathogenic *Escherichia coli* (UPEC) causes up to 80% of community-acquired UTIs (55, 56). UPEC filamentation is essential for the continuous generations of intracellular bacterial community formation and, ultimately, the establishment of quiescent intracellular reservoirs (41, 57).

Although some groups investigate the use of RecA as a therapeutic target, this protein plays multiple roles in general DNA maintenance, and interference with RecA function has pleiotropic effects in commensal organisms. In addition to the multifactorial role of the SOS response during infection, there are multiple lines of evidence indicating that SulA is a prime antivirulence target, particularly for the treatment of UTIs. For example, the absence of SOS regulatory components and DNA repair enzymes attenuates UTI (58, 59). In addition, *sulA* expression was directly visualized within bladder epithelial cells during UTI. UTI is attenuated in the absence of SulA, and the establishment of quiescent intracellular reservoirs relies on the production of SulA (41, 51, 52). Taken together, these observations led us to identify compounds that inhibit SulA activity as potential adjunct or independent therapeutic agents for UTI. In this study, we determined the efficacy of four medicinal food products (i.e., pineapple juice, blueberry juice, sodium bicarbonate, and cranberry juice) commonly used to treat UTI in the prevention of UPEC filamentation. Pineapple juice and blueberry juice had no effect on SulA-mediated filamentation. We determined that sodium bicarbonate induced a significant increase in UPEC filamentation, while cranberry juice significantly reduced UPEC filamentation. We further demonstrate that subfractions of cranberry retain the anti-filamentation efficacy for clinical UPEC isolates. This study provides the first mechanistic evidence for the efficacy of cranberry juice in the treatment of UTI.

## MATERIALS AND METHODS

### Bacterial strains

UTI89 is a well-characterized prototypical UPEC strain that was obtained from a patient with cystitis (60). CFT073 is a well-characterized UPEC strain that was obtained from a patient with pyelonephritis (61). BL21 Δ*lon* (Thermo-Fisher, Waltham, MA) was transduced with a P*ara* plasmid carrying the *sulA* gene amplified from UTI89 (P_ara_::*sulA_UPEC_*) (13). The primers used for cloning and sequencing of *sulA* are detailed in Table 1. UPEC clinical isolates, including PEDSUTI011, were obtained from children presenting for treatment of UTI at Nationwide Children’s Hospital and were denoted as non-febrile (cystitis) or febrile (pyelonephritis) (62).

**TABLE 1:** Primers^*a*^

Primer name	Sequence
5PEcoSDsulA	CCG **GAA TTC** AGG AGG TTG ATT ATG TAC ACT GCA TAT GCA CA
3PsulAH3	CCC **AAG CTT** TAC TTA ATG ATA CAA ATT AGA GTG AAT TTT TAG CCC GG
pBAD33 forward sequencing	ATG CCA TAG CAT TTT TAT CC
pBAD33 reverse sequencing	CCT GAT ACA GAT TAA ATC

^*a*^ The primer sequences were determined using ADDGENE (addgene.org). The engineered restriction enzyme recognition sites are bolded.

### Bacterial cultivation for induction of filamentation by activated phagocytes

UPEC strains were grown overnight statically at 37°C in Luria Broth (LB) (Fisher Scientific, Waltham, MA). Overnight LB cultures were diluted 1:1,000 into M63 minimal media containing 0.1% casamino acids (Fisher Scientific), 0.3% glycerol (Fisher Scientific), 1 mM MgSO_4_, and 0.1 mg/mL thiamine HCl (Fisher Scientific) and were grown aerobically (225 revolutions per min) at 37°C for 8 h until early stationary phase was reached (OD_600_ = ~0.8). Early stationary phase cultures of strains grown in M63 media were diluted 10-fold in phosphate-buffered saline (PBS; Sigma Chemicals, St. Louis, MO) and frozen at −80°C in 45% glycerol as starter cultures for minimal media experiments. For co-culture assays, frozen stocks of early stationary phase cultures of UPEC strains grown in Dulbecco’s modified Eagle’s medium (DMEM; Cellgro, Manassas, VA) supplemented with 10% heat-inactivated fetal bovine serum (FBS; Invitrogen, Carlsbad, CA) (DMEM-S) were prepared as previously described (42). The growth rate of strains grown in M63 media or DMEM-S was monitored by measurement of the optical density at 600 nm at 30-min intervals for 12 h on a Synergy HT microplate reader (Agilent, Santa Clara, CA).

### Induction of filamentation with activated phagocytes

Induction of filamentation of the clinical UPEC isolates was performed as previously described (42). Briefly, approximately 1 h prior to harvest of the phagocytes, early stationary phase cultures of UPEC strains grown in DMEM-S were thawed, diluted 1:10 into pre-warmed DMEM-S, and were grown at 37°C in 5% atmospheric CO_2_ for one hour in 12 × 75 mm^2^ polystyrene round bottom tubes (Becton Dickinson, Franklin Lakes, NJ). Approximately 5 × 10^5^ THP-1 lipopolysaccharide-activated phagocytes (Thermo Fisher Scientific, Waltham, MA) were infected with bacterial strains at an initial multiplicity of infection (MOI) of 1 (5 × 10^5^ UPEC) in 500 µL of DMEM-S at 37°C in 5% atmospheric CO_2_. After 2 h of co-culture, phagocytes were lysed, the samples were fixed with paraformaldehyde, and bacterial morphology was determined by flow cytometric analysis on an LSR II cytometer (Becton Dickinson, Franklin Lakes, NJ) as previously described (42).

### Bacterial cultivation for ectopic induction of SulA-mediated filamentation

BL21 Δ*lon* (P_ara_::*sulA_UPEC_*) was grown aerobically in an M9 medium containing 0.5% glycerol at 37°C, 225 revolutions per min. For supplementation with juice, a 2× M9 stock solution was supplemented with the juice concentration indicated, the final volume was increased with purified water, and then the medium was filter sterilized. The juices were commercially available: Pineapple (R.W. Knudsen Organic Juice), Blueberry (R.W. Knudsen Organic Juice), diet cranberry juice cocktail (Ocean Spray Cranberries, Inc), and 100% PURE cranberry (Ocean Spray Cranberries, Inc). Sodium bicarbonate (Fisher Scientific, Waltham, MA) was added to a final concentration of 3% (wt/vol). To determine the contribution of various cranberry components, three independent fractionated cranberry powders (see below) were used at the concentrations presented in the text. After 2 h of growth, parallel cultures were grown in the presence or absence of d-arabinose (Fisher Scientific, Waltham, MA) to the final concentration indicated in the text for induction of filamentation in the screening strain BL21 lon P_ara_::*sulA_UPEC_*. In addition, we ensured that the concentration of Mitomycin C used did not induce bacterial lysis for each of the clinical strains.

Growth was monitored by measuring changes in culture density using the absorbance at 600 nm. Filamentation was quantified using two different microscopic approaches. Cultures were dried on glass slides, heat fixed, and stained with Gram stain (Fisher Scientific, Waltham, MA). For each slide, a minimum of 10 random fields were imaged on an Axio Lab.A1 light microscope using an Axiocam ERc 5s color camera (Carl Zeiss Inc., Thornwood, NY). For each field, the length of each filament was determined using the segmented line measurement tool in ImageJ (NIH), and a minimum of 100 bacterial cells were measured for each biological replicate. For filamentation rate, the number of filaments (>16 µm) and the number of bacillary morphotypes were counted for each random field. The length determination for a filament is based on prior studies indicating that 10 µm is necessary for resistance from phagocytosis and the next closest number that accounts for the doubling rate of growth (2^4^) and is a size that is consistent for determination of filamentation in the field (13, 38, 43, 44). The rate was then determined as the percentage of total bacterial cells counted. Each experimental condition was evaluated on a minimum of three independent occasions for each bacterial strain.

### Induction of SulA-mediated filamentation with mitomycin C

CFT073 or PEDSUTI011 were grown in an M9 containing 0.5% glycerol aerobically (225 revolutions per min) at 37°C for 2 h. Parallel cultures were either untreated or induced with 200 or 400 ng/mL mitomycin C, respectively (Fisher Scientific, Waltham, MA), and the growth was continued for an additional 4 h in the presence or absence of cranberry constituents as indicated above and in the text.

### Fractionation of cranberries

Three different subfractions of whole cranberries were provided in powdered form and were prepared using a modified method reported by Howell et al. (63). Juice samples from mash processing went through C18 SepPak cartridges (Waters Corps., Milford, MA). The cartridges were first washed with 100% deionized water and then 15% methanol (water:methanol 85:15 vol/vol). Eluents were discarded. The polyphenolic fraction was further eluted from 100% methanol and then went through Sephadex LH-20 (Sigma Chemical Co., St. Louis, MO). Deionized water was used as the starting mobile phase, and eluents were discarded. The column was washed with 25% ethanol to obtain fractions rich in anthocyanins (Fractions B and C in Table 2). Fractions rich in proanthocyanidins (PACs) were further washed with 70% acetone (Fraction A in Table 2). Fractions were placed on a rotary evaporator to remove solvent and subsequently freeze-dried for further analysis. The composition of the cranberry fractions was determined using multiple approaches. Anthocyanins were analyzed by HPLC as previously described (64). PACs were analyzed using the colorimetric method (BL-DMAC) (65) with PAC-A2 as a reference standard and were also quantified using a modified colorimetric method (OSC-DMAC) (66). Total phenolics were measured by Folin as previously described (67). The powdered cranberry constituents were added to fresh M9 0.5% glycerol medium to the final concentrations indicated and filter sterilized. The initial concentration of powder used was based on estimates from the concentrations of the PURE cranberry juice that demonstrated efficacy and back calculated from the amount of cranberries used in the purification process. The final concentrations were empirically determined by serial dilution.

**TABLE 2:** Composition of the cranberry extracts based on dry weight^*a*^

Sample name	Powder A	Powder B	Powder C	Pure juice
Total anthocyanins (mg/g)	0.73	113.21	115.27	0.18
Anthocyanin profile^*c*^				
Cyanidin-3-arabinoside	0.45	26.30	25.17	0.03
Cyanidin-3-galactoside	0.05	27.39	23.28	0.05
Cyanidin-3-glucoside	0.01	2.79	2.32	<0.01
Peonidin-3-arabinoside	0.15	22.66	26.31	0.02
Peonidin-3-galactoside	0.05	28.02	31.22	0.07
Peonidin-3-glucoside	0.02	6.05	6.97	0.01
Total organic acids (%)	0.04	0.25	0.08	2.43
PACs BL DMAC dwb^*b*^ (mg/g)^*d*^	241.59	96.82	81.08	0.40
PACs OSC DMAC dwb^*b*^ (mg/g)^*e*^	943.74	79.10	60.48	1.18
Total phenolics by folin (mg/g)^*f*^	856.03	720.58	743.83	2.02

^*a*^ Abbreviations: PAC, proanthocyanidin; DMAC, 4-dimethylaminocinnamaldehyde; dwb, dry weight based.

^*b*^ PACs reported in extracts A, B, and C are based on dry weight.

^*c*^ Anthocyanins were analyzed by HPLC (64).

^*d*^ PACs were also analyzed using the colorimetric method (BL-DMAC) (65) with PAC-A2 as a reference standard.

^*e*^ PACs were quantified using a modified colorimetric method (OSC-DMAC) (66).

^*f*^ Total phenolics were measured by Folin (67).

## RESULTS

### UPEC clinical isolates display filamentation during co-culture with activated phagocytes

**Fig 1:**
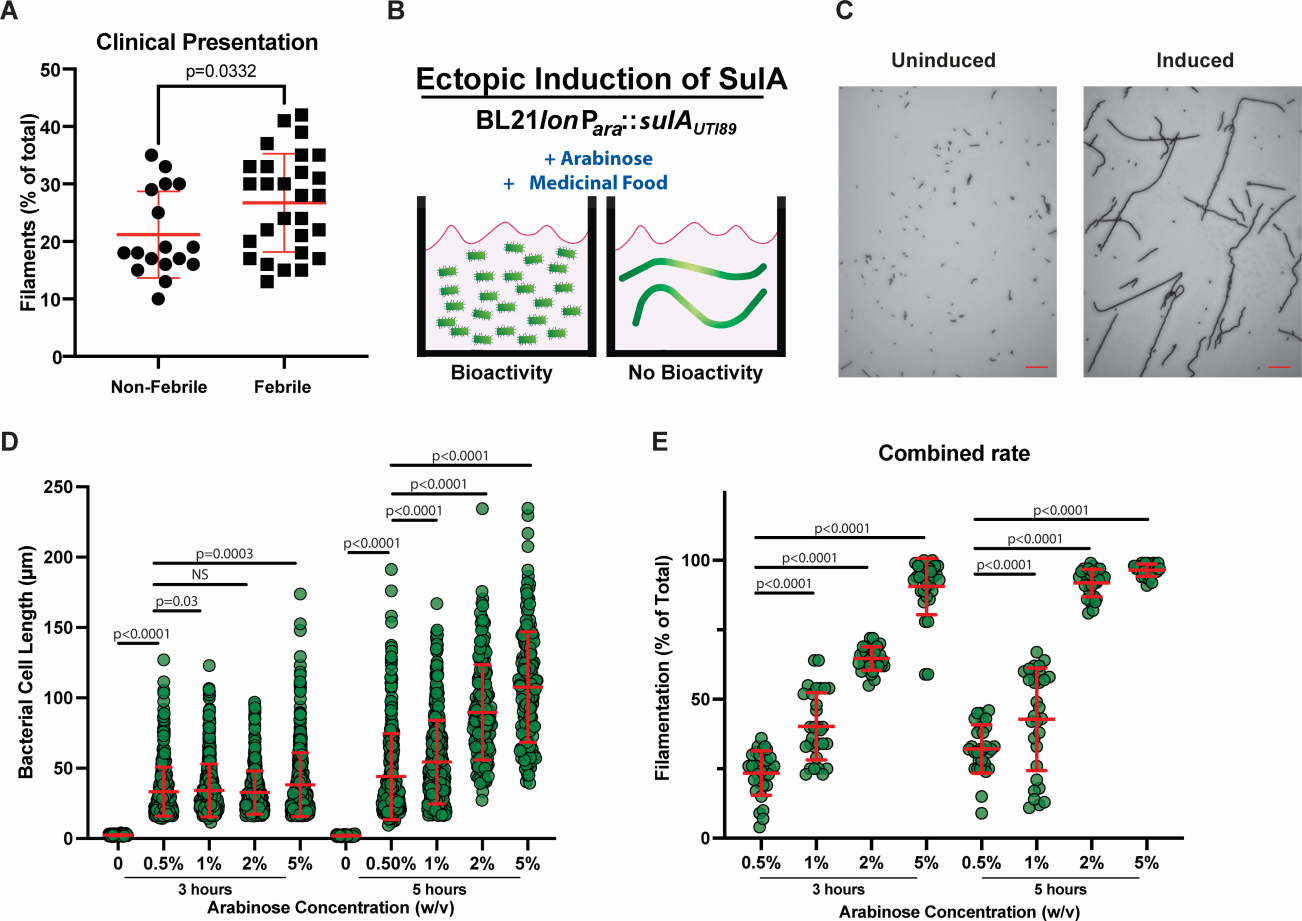
The overall bacterial cell length and rate of filamentation of BL21 Δ*lon* (P_ara_::*sulA_UPEC_*) is dependent on time and arabinose concentration. (**A**) UPEC isolates in early logarithmic growth phase were co-cultured in the presence or absence of fresh activated primary phagocytes (MOI 1:1) for 2 h and analyzed as described in Materials and Methods. Quantification of the extent of filamentation is presented as the mean of duplicate samples and replicated on three independent occasions. Isolates were categorized based on the clinical diagnosis of UTI into non-febrile and febrile. Statistical significance was determined by an unpaired two-tailed Student’s *t*-test (GraphPad Prism). (**B**) Schematic representation of the phenotypes in the presence and absence of molecules with anti-filamentation properties. (**C**) BL21 Δ*lon* (P_ara_::*sulA_UPEC_*) was grown in M9 minimal media containing 0.5% glycerol. After 2 h of growth, the final concentrations of arabinose indicated were added, and the growth continued for 3 or 5 h. Representative images of BL21Δ*lon* (P_ara_::*sulA_UPEC_*) in the absence (uninduced) and presence (induced) of 5% arabinose after 5 h of growth. Red scale bar = 10 µm. (**D**) Individual bacterial lengths were measured in the presence of indicated concentrations of arabinose. Each data point represents the length of a single bacterium as measured in ImageJ as described in Materials and Methods. (**E**) The number of filamentous morphotypes (<16 µm) is reported as a percentage of the total bacteria in each of 10 random fields. Each experiment was performed using 10 random fields of view on three independent occasions (*n* = 30). The mean value ± standard deviation is indicated in red. Statistical significance was determined by unpaired, two-tailed Student’s *t*-test (GraphPad Prism).

We first sought to determine the conservation of filamentation in multiple clinical UPEC isolates under host-derived conditions. We previously demonstrated that co-culture with either cultured or primary phagocytes induces UPEC filamentation *in vitro* (42). Flow cytometry provides a highly sensitive and quantitative approach to enumerate the different morphologies in a population (42). Under typical culture conditions of UPEC in LB broth, we observe a strong correlation (*R*^2^ = 0.992) of the number of bacillary bacteria enumerated by flow cytometry and by traditional colony-forming units (CFU) on LB agar (Fig. S1). To determine whether filamentation reveals a common mechanism among UPEC strains, we analyzed 42 UPEC isolates from our pediatric UPEC collection (62). Each isolate was co-cultured with activated THP-1 phagocytes for 3 h, and the extent of filamentation was measured by flow cytometry as described in Materials and Methods. Filamentation was observed in all clinical isolates, indicating that morphological plasticity is a conserved response to the antimicrobial agents produced by activated phagocytes. To determine whether the extent of filamentation of the population correlated with the clinical disease severity, the isolates were segregated based on a clinical diagnosis of cystitis (non-febrile) or pyelonephritis (febrile) at the time of specimen collection (Fig. 1A). A statistically significant increase was observed in the rate of filamentation for febrile isolates as compared to non-febrile isolates.

### Dose-dependent filamentation of UPEC during ectopic production of SulA

Our published data indicate that prevention of filamentation, through inactivation of *sulA*, is sufficient to promote clearance of UPEC (41, 42). We proposed to mimic this phenotype through the pharmacological inhibition of SulA-mediated filamentation. In order to specially identify molecules that inhibit the FtsZ-SulA complex (13–16, 68), a screening strain was constructed in the laboratory *E. coli* strain BL21 (Fig. 1B). Overproduction of SulA_UPEC_ was performed using a multicopy plasmid containing *sulA* from a prototypical cystitis UPEC strain, UTI89, under tight control of the arabinose promoter [BL21(P_ara_::*sulA_UPEC_*)] (13, 69). Finally, to prevent recovery from filamentation and increase the rigor and reliability of the results, the strain lacks the Lon protease (42, 70). The assay was designed in a laboratory strain of *E. coli* where ectopic induction of the SulA ortholog results in a significant increase in bacterial cell length to test the ability of pharmacological inhibitors to restore cell division. The inhibition of SulA with a pharmacological inhibitor is a dominant phenotype, allowing screening of complex pools of compounds to evaluate efficacy. Candidate molecules will then be validated in pathogenic UPEC strains under induction of the intact SOS response.

The induction of filamentous morphotypes in BL21Δ*lon* (P_ara_::*sulA_UPEC_*) was both time- and dose-dependent on the concentration of arabinose. Representative images of induced (5% arabinose for 5 h) and uninduced cultures are presented in Fig. 1C. Arabinose concentrations from 0.5% to 5% were evaluated by measuring individual bacterial cell lengths and the rate of filamentation at 3 and 5 h post-induction. In the absence of arabinose, all bacteria were less than 4.1 µm in cell length at both time points (Fig. 1D). The addition of 0.5% arabinose resulted in a significant increase in overall cell length (16–40 µm; mean 33.4 µm) in 24% (mean) of the population at 3 h and increasing in overall cell length (16–181 µm; mean 44 µm) in 32% (mean) of the population at 5 h (Fig. 1D and E). The addition of 5% arabinose resulted in a significant increase in the range of overall cell lengths (16–174 µm; mean 38 µm) at 3 h and an additional increase in overall cell lengths (40–235 µm; mean 108 µm) in up to 97% of the population (Fig. 1D and E) by 5 h. Since 0.5% arabinose provided significant induction of both the overall bacterial cell length and the rate of filamentation at 5 h, these conditions were selected for further screening to identify molecules that would significantly reduce filamentation.

### Medicinal foods alter the extent of UPEC filamentation

**Fig 2:**
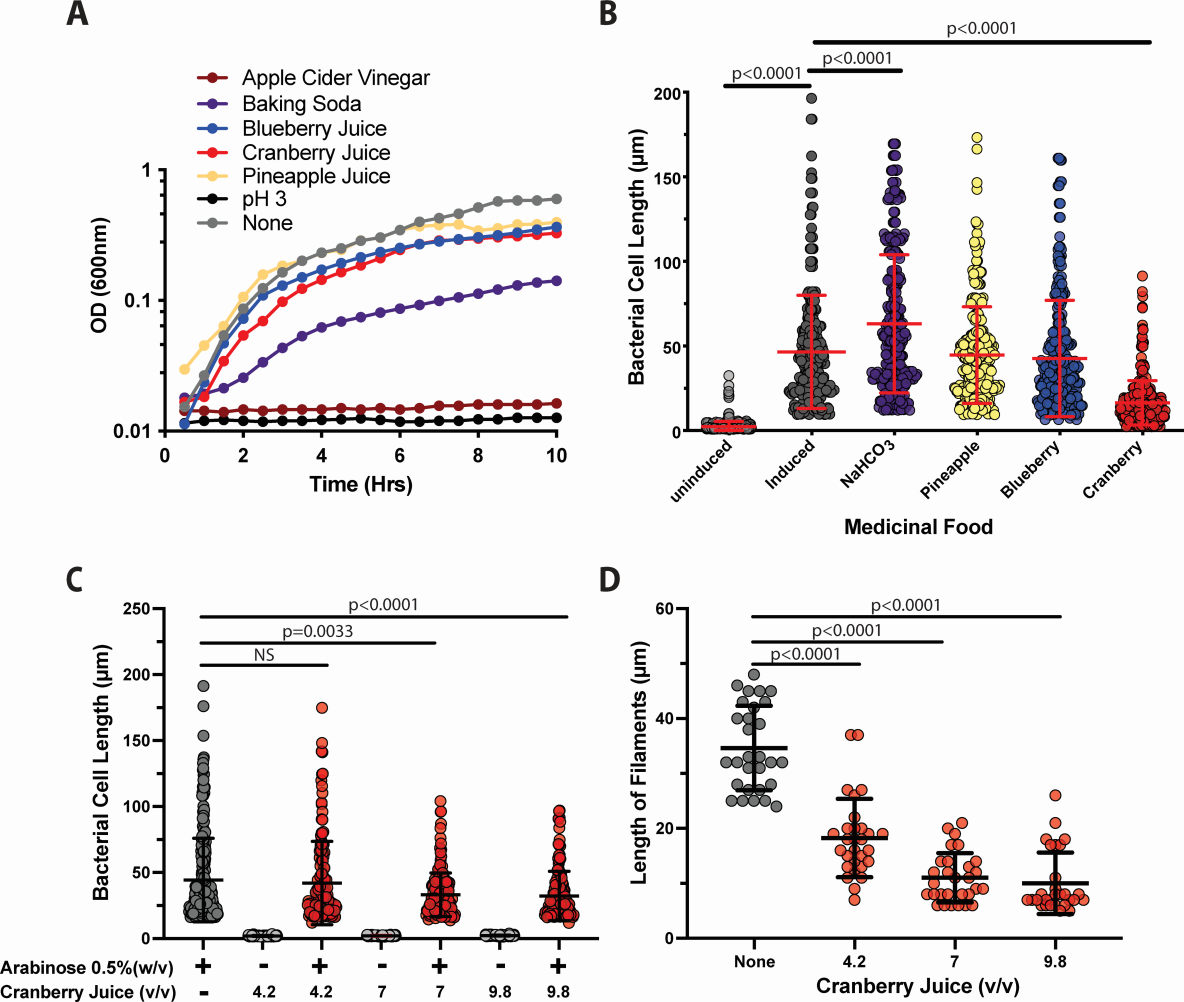
The effect of medicinal food products on cell length and rate of filamentation of BL21 Δ*lon* (P_ara_::*sulA_UPEC_*). BL21 Δ*lon* (P_ara_::*sulA_UPEC_*) was grown in M9 minimal media containing 0.5% glycerol and the medicinal food indicated. Baking soda (NaHCO_3_) was added to 3% (wt/vol), pineapple juice, blueberry juice, and diet cranberry juice were added to a final concentration of 10% (vol/vol). After 2 h of growth, parallel cultures with or without arabinose supplementation were placed to a final concentration of 0.5%, and the growth was continued for 5 h to induce filamentation. (**A**) Optical density (OD) at 600 nm was determined every 30 min. BL21 Δ*lon* (P_ara_::*sulA_UPEC_*) was grown in M9 minimal media containing 0.5% glycerol and the indicated medicinal food. (**B**) Individual bacterial lengths were measured in the presence of the indicated medicinal food product as described in Fig. 1. (**C**) BL21 Δ*lon* (P_ara_::*sulA_UPEC_*) was grown in M9 minimal media containing 0.5% glycerol containing the final concentrations of the PURE unsweetened 100% cranberry juice indicated. After 2 h of growth, arabinose was added to a final concentration of 0.5%, and the growth was continued for 5 h. Individual bacterial cell lengths were measured as described in Fig. 1. (**D**) The rate of filamentation was determined as described in Fig. 1. Each experiment was performed using 10 random fields of view on three independent occasions. The mean value ± standard deviation is indicated in red or black (for ease of visualization). Statistical significance was determined by unpaired, two-tailed Student’s *t*-test (GraphPad Prism).

We first evaluated the effect of natural medicinal foods commonly used for treatment of UTI on SulA_UPEC_-mediated filamentation. Pineapple juice has been recommended to aid in the resolution of UTI due to the presence of the enzyme bromelain that may enhance absorption of antibiotics (71). Consumption of blueberry and cranberry juices has been suggested to aid in the resolution of UTI due to the high concentrations of nutraceuticals and vitamin C (72–75). Sodium bicarbonate is recommended to regulate the pH of the urine to reduce the burden of disease (76, 77). The use of vinegar has long-standing use in homeopathic medicine and has been evaluated for efficacy in treating catheter-associated UTI (78–81). The screening strain BL21 Δ*lon* (P_ara_::*sulA_UPEC_*) was grown in the presence or absence of each of the medicinal foods to determine any potential effect on growth (Fig. 2A). We observed that there was a nominal effect of the addition of the medicinal food on the overall growth rate of BL21 Δ*lon* (P_ara_::*sulA_UPEC_*) with the exception of apple cider vinegar, which abolished growth. The growth was similar to that in media adjusted to pH 3, the pH of the media containing apple cider vinegar. Due to the growth defect, apple cider vinegar was not evaluated further. For the remaining medicinal foods, BL21 Δ*lon* (P_ara_::*sulA_UPEC_*) was grown at the indicated concentration for 2 h then filamentation was induced with the addition of 0.5% arabinose to the media for an additional 5 h. The medicinal food was present for the duration of the experiment. The addition of 10% pineapple juice or 10% blueberry juice had no effect on the overall bacterial lengths of strain BL21 Δ*lon* (P_ara_::*sulA_UPEC_*) (Fig. 2A). However, the addition of 3% sodium bicarbonate significantly increased the overall bacterial lengths (12–170 µm) with a mean value of 63.1 µm as compared to overall bacterial cell lengths (10–196 µm) with a mean of 46.8 µm in the presence of arabinose alone. The addition of 10% cranberry juice cocktail significantly reduced the overall cell length (2–91 µm) with a mean length of 16 µm. Thus, of the four conditions evaluated, we observed that only the diet cranberry juice cocktail significantly reduced filamentation (Fig. 2B).

### PURE cranberry juice prevents SulA-mediated filamentation

We initially selected the diet cranberry cocktail as this was the common formulation used by patients due to the absence of added sugars (82–86). Although we detected a significant difference in the extent of filamentation in the presence of cranberry juice cocktail, we could not exclude the potential for effects from other components of the cocktail (e.g., flavorings and artificial sweeteners). Therefore, we chose to evaluate the efficacy of PURE unsweetened 100% cranberry juice in the prevention of filamentation. We observed a dose-dependent reduction in arabinose-induced bacterial cell length from 16–191 µm (mean 44 µm) to 11.9–97 µm (mean 32 µm) in the presence of 9.8% pure cranberry juice (Fig. 2C). There was also a significant decrease in the rate of filamentation from 34% in the absence of cranberry to 10% in the presence of the highest concentration of pure cranberry juice (9.8%; Fig. 2D). Cranberry juice alone had no effect on the overall bacterial cell length in the absence of arabinose (Fig. 2C and D)

### Cranberry constituents prevent SulA-mediated filamentation

**Fig 3:**
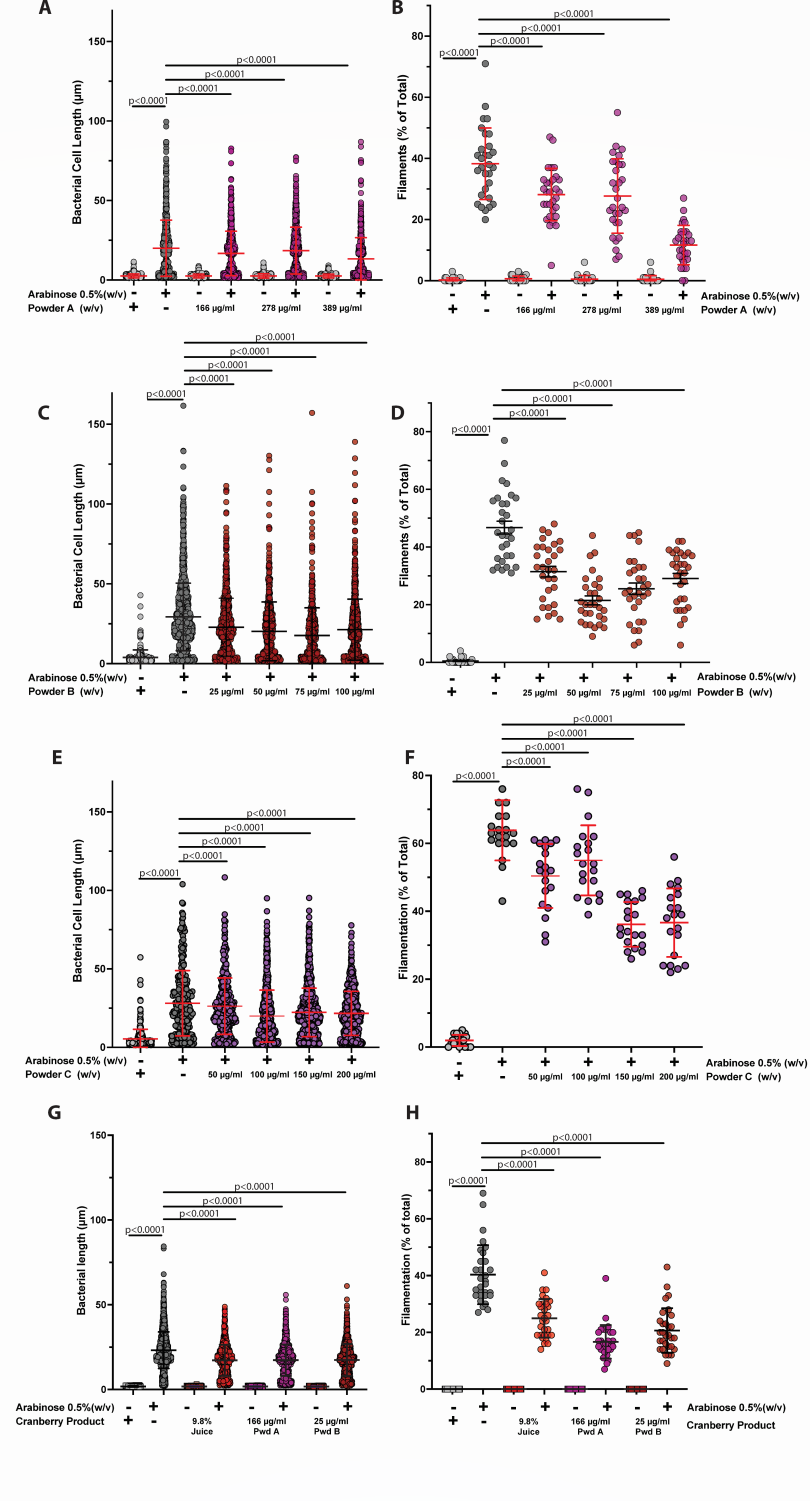
The effect of cranberry powders on overall cell length and rate of filamentation of BL21 Δ*lon* (P_ara_::*sulA_UPEC_*). BL21 Δ*lon* (P_ara_::*sulA_UPEC_*) was grown in M9 minimal media containing 0.5% glycerol containing the final concentrations of either powder A, powder B, or powder C as indicated. After 2 h of growth, arabinose was added to a final concentration of 0.5%, and the growth continued for 5 . (**A, C, E, G**) Individual bacterial cell lengths were measured as described in Fig. 1. (**B, D, F, H**) The rate of filamentation was determined as described in Fig. 1. Each experiment was performed using 10 random fields of view on three independent occasions (*n* = 30). The mean value ± standard deviation is indicated in red or black (for ease of visualization). Statistical significance was determined by unpaired, two-tailed Student’s *t*-test (GraphPad Prism).

To further delineate the components of the cranberry that are effective in the prevention of bacterial filamentation, subfractions of cranberries were evaluated using the BL21 Δ*lon* (P_ara_::*sulA_UPEC_*) screening strain in the presence or absence of arabinose. Each powder was titrated to determine the optimal concentrations that demonstrate potential efficacy in the prevention of SulA-mediated filamentation. The investigators were blinded to the composition of the cranberry fractions to ensure the rigor and reliability of the results; however, we were provided target concentrations to initially test based on the efficacy of the 9.8% pure cranberry juice. For powder A, we observed a modest but statistically significant dose-dependent decrease in the mean bacterial cell length (20 vs 13.3 µm) at the maximum concentration tested (389 µg/mL) as compared to the no cranberry control (Fig. 3A). The rate of filamentation was significantly reduced from 38% to 12% (Fig. 3B). For powder B, we observed effects in the reduction of both the overall cell length and rate of filamentation (Fig. 3C and D). We observed that the mean cell length was most reduced in the presence of 75 µg/mL powder B (30 vs 17 µm) compared to the no cranberry control. The reduction in the rate of filamentation was the most pronounced in the presence of 50 µg/mL powder B (47% vs 21%) compared to the absence of cranberry. For powder C, a statistically significant and dose-dependent decrease was observed in the overall cell length from 28 µm in the absence of powder C to 20 µm in the presence of 200 µg/mL powder C (Fig. 3E). A dose-dependent and statistically significant decrease was also observed in the rate of filamentation from 64% to 36% in the absence and presence of powder C, respectively (Fig. 3F). Thus, multiple fractions of cranberry retain the ability to inhibit SulA-medicated filamentation.

The cranberry extracts were initially tested independently to evaluate multiple concentrations and determine dose dependence. The efficacies of the cranberry extracts were then directly compared at the concentrations with maximal efficacy. At the concentrations tested, a similar statistically significant decrease in the overall cell length was observed for the pure cranberry juice, powder A, and powder B (17.2, 17.3, and 17.4 µm, respectively) as compared in the absence of cranberry product (23.1 µm) (Fig. 3G). We did observe differences in the rate of filamentation between the three cranberry products tested (24.9%, 16.7%, and 20.7%) as compared to the absence of cranberry (41%) (Fig. 3H). Due to the timing of receiving powder C, we did not have sufficient amounts of powder A to provide a full comparison of all the fractions within the same experiment, an unavoidable limitation of this study. Thus, although different concentrations were used, the three different sources of cranberry demonstrated similar efficacy in the reduction of SulA-mediated filamentation.

### Cranberry constituents reduce filamentation of clinical isolates under native induction of SulA

**Fig 4:**
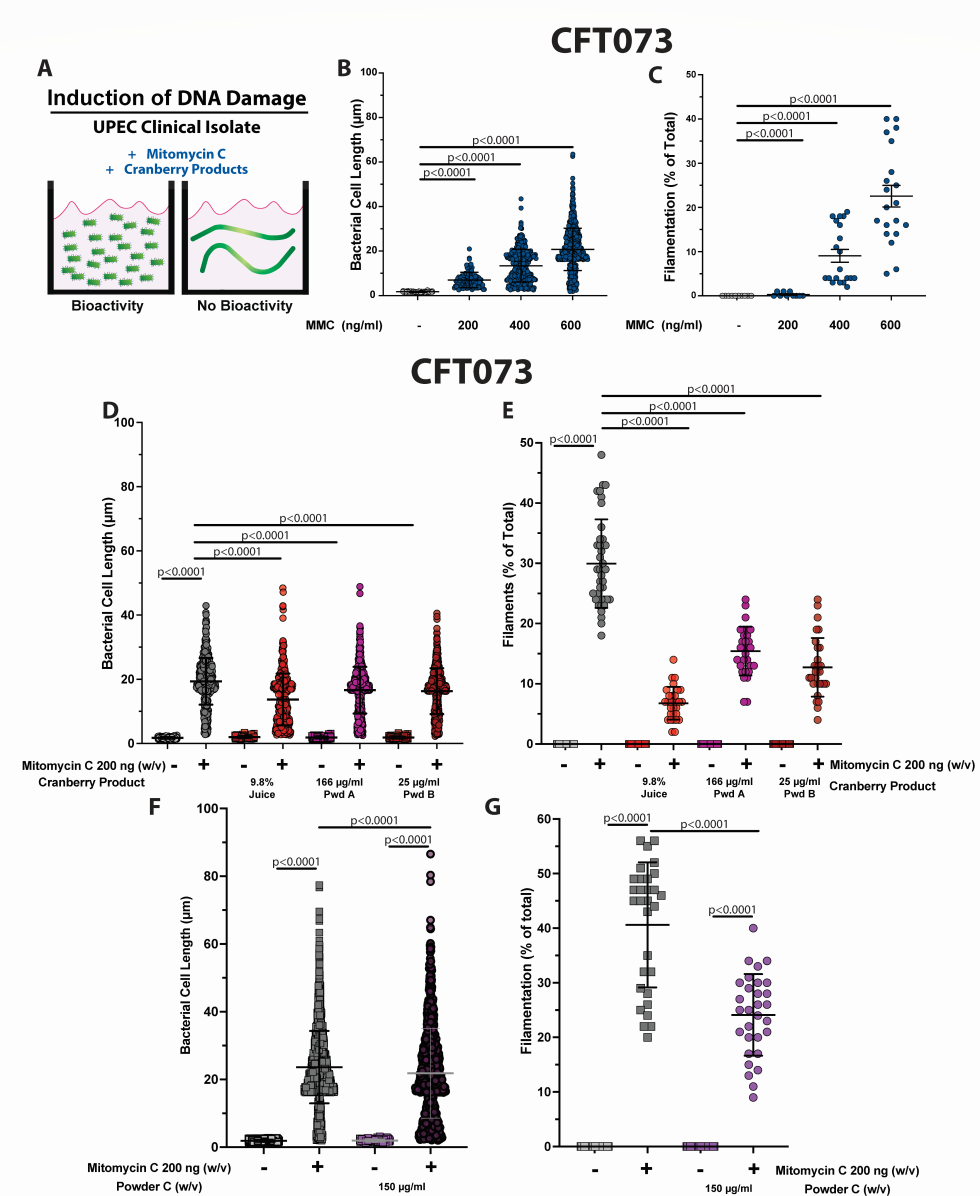
The effect of PURE cranberry juice, powder A, powder B, and powder C on cell length and rate of filamentation of CFT073. (**A**) Schematic representation of the phenotypes in the presence and absence of molecules with anti-filamentation properties. (**B, C**) CFT073 was grown in M9 minimal media containing 0.5% glycerol. After 2 h of growth, the DNA intercalating agent, mitomycin C was added to the final concentrations indicated, and the growth continued for 5 h. (**D–G**) CFT073 was grown in M9 minimal media containing 0.5% glycerol containing the final concentrations of pure cranberry juice, powder A, powder B, or powder C as indicated. After 2 h of growth, mitomycin C was added to a final concentration of 200 ng/mL, and the growth continued for 5 h. (**B, D, F**) Individual bacterial cell lengths were measured as described in Fig. 1. (**C, E, G**) The rate of filamentation was determined as described in Fig. 1. Each experiment was performed using 10 random fields of view on three independent occasions (*n* = 30). The mean value ± standard deviation is indicated in black or gray (for ease of visualization). Statistical significance was determined by unpaired, two-tailed Student’s *t*-test (GraphPad Prism).

Pathogenic strains of *E. coli* can behave differently than laboratory *E. coli* strains due to the larger genomic content. In addition, induction of the intact SOS response may also modulate responses to potential therapeutics. Thus, it is important to evaluate the efficacy in uropathogenic clinical isolates to validate the mechanism (Fig. 4A). The chemical agent mitomycin C introduces DNA lesions that induce the DNA damage repair response and SulA-mediated filamentation (13, 87, 88). To determine the efficacy of cranberry constituents to prevent filamentation in intact UPEC strains, SulA-mediated filamentation was induced in CFT073 (a prototypical and highly studied pyelonephritis isolate) with increasing concentrations of mitomycin C. Both the overall bacterial cell length and the rate of filamentation were concentration dependent (Fig. 4B and C). As expected, we did not observe the same overall increase in cell length as we observed using ectopic production of SulA in the laboratory strain BL21 Δ*lon* (P_ara_::*sulA_UPEC_*) due, in part, to the repair of DNA damage and presence of the Lon protease. Powders A and B both significantly reduced the overall cell length from 20 to 18 µm. The greatest decrease in the overall cell length was observed in the presence of 9.8% pure cranberry juice (15 µm; Fig. 4D). A statistically significant decrease in the rate of filamentation was also observed in CFT073 from 30% to 6%, 15%, and 10% for the pure cranberry juice, powder A, and powder B, respectively (Fig. 4E). As with the screening strain, we also observed a significant decrease in the overall bacterial cell length from 23 to 20 µm in the presence of powder C (Fig. 4F). The rate of filamentation was also reduced from 40% to 25% in the presence of powder C (Fig. 4G). Thus, the ability of cranberry products to reduce filamentation was retained during induction of the intact SOS response in a prototypical uropathogen.

**Fig 5:**
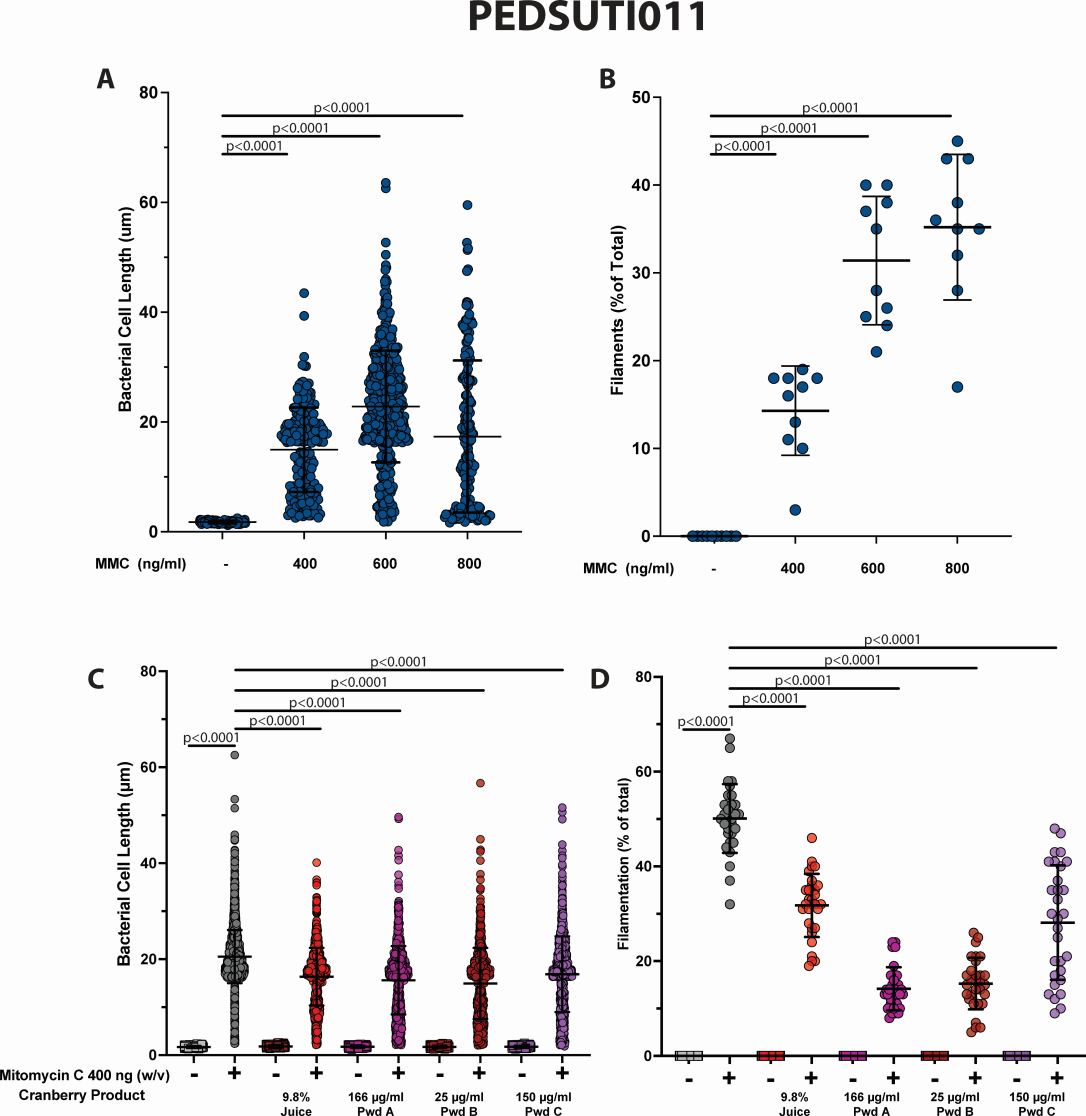
The effect of PURE cranberry juice, powder A, powder B, and powder C on cell length and rate of filamentation of PEDSUTI011. PEDSUTI011 was grown in M9 minimal media containing 0.5% glycerol containing the final concentrations of pure cranberry juice, powder A, powder B, or powder C as indicated. After 2 h of growth, mitomycin C was added to a final concentration of 400 ng/mL, and the growth continued for 5 h. (A, C) Individual bacterial cell lengths were measured as described in Fig. 1. (B, D) The rate of filamentation was determined as described in Fig. 1. Each experiment was performed using 10 random fields of view on three independent occasions (*n* = 30). The mean value ± standard deviation is indicated in black. Statistical significance was determined by unpaired, two-tailed Student’s *t*-test (GraphPad Prism).

To determine the efficacy of cranberry constituents to prevent filamentation in another clinical UPEC isolate, the SOS response was induced in PEDSUTI011 (a clinical isolate from a child with a febrile infection) with increasing concentrations of mitomycin C. Both the overall bacterial cell length and the rate of filamentation were dependent on the concentration of mitomycin C, although the empirically determined strain-specific concentration of mitomycin C was different than that used for CFT073 (Fig. 5A and B). The pure cranberry juice, powder A, powder B, and powder C significantly reduced the overall cell length from 21 to 16, 15, 15, and 17 µm, respectively (Fig. 5C). A statistically significant decrease in the rate of filamentation was also observed in PEDSUTI011 from 50% to 32%, 14%, 15%, and 28% for the pure cranberry juice, powder A, powder B, and powder C, respectively (Fig. 5D).

### Analysis of the cranberry extracts used in this study

As all extracts were effective in both reducing filament length and the rate of filamentation, a targeted analysis of extract components was performed to begin to identify the potential active molecules. PACs were the most enriched in powder A, where higher concentrations of the extract were required for efficacy. Powders B and C were enriched for anthocyanins as compared to the PURE juice and powder A (Tables 2 and 3). There were similar levels of total phenolics in all three powders. When normalizing for the amount of powder/juice included in the medium, there is no clear compound that appears to be equivalent in this analysis, suggesting that there are other components not measured here that contribute to the phenotype. Alternatively, more than one component could contribute to the interference with SulA-mediated filamentation.

**TABLE 3:** Calculated compositions of cranberry treatments in prepared bacterial medium in the observed active dose^*a*^

Sample name	Powder A	Powder B	Powder C	Pure juice
Total anthocyanins (mg/mL)	0.12	2.83	17.29	0.02
Total organic acids (mg/mL)	0.07	0.06	0.12	2.38
PACs BL DMAC (mg/mL)	40.10	2.42	12.16	0.04
PACs OSC DMAC (mg/mL)	156.66	1.98	9.07	0.12
Total phenolics by folin (mg/mL)	142.10	18.01	111.57	0.20

^*a*^ Powder A composition shown was based on 166 µg/mL; powder B composition shown was based on 25 µg/mL; powder C composition shown was based on 150 mL; and pure was based on 9.8% (98 µl/mL).

## DISCUSSION

The rise in antibiotic-resistant organisms has created a pressing need to develop novel antimicrobials. The average cost for the development of an antimicrobial can reach billions of dollars. Ultimately, targeting virulence traits that are shared by multiple pathogenic strains will increase the utility of a potential therapeutic. Filamentous morphotypes are readily observed in urine samples obtained from patients who suffer from UTI caused by UPEC, *Klebsiella pneumoniae*, *Enterobacter aerogenes*, and *Proteus mirabilis* (53). In addition, filamentous morphotypes are observed within human samples or in preclinical models of infection for *Haemophilus*, *Salmonella*, *Legionella*, *Yersinia*, *Mycobacterium*, and *Citrobacter* (18, 39, 40). Moreover, we have identified SulA-dependent filamentation as an essential virulence trait of both UPEC and nontypeable *Haemophilus influenzae* (41, 42, 51, 89). In addition, the SulA-FtsZ protein interaction that controls filamentation is highly conserved, further suggesting that agents that prevent UPEC filamentation will also be effective against other pathogens. As with any therapeutic, there is the potential for bacterial adaptation to escape the new treatment. However, mutations that prevent cranberry components from binding to SulA would also prevent the SulA-FtsZ protein interactions. Thus, the bacteria would remain in the bacillary morphology and be susceptible to phagocyte-mediated killing and antimicrobial agents that act on the cell division machinery. In addition, targeting of the filamentous morphotypes due to the induction of SulA should also help reduce the accumulation of antibiotic-resistant strains through enhanced clearance of the individual bacteria that are capable of error-prone DNA damage repair through the SOS response.

Prophylaxis with cranberry juice and cranberry capsules is effective to prevent recurrent UTIs in young and middle-aged women (90–93). In addition, a recent Cochrane systematic review of studies involving almost 9,000 patients concluded that there is clinical evidence for reduced rates of symptomatic culture-proven recurrent UTIs in women and children who consume cranberry products (75). However, there is no current consensus on the mechanism of efficacy nor the specific components of cranberries that are efficacious. Many labs have implicated PACs in preventing binding of UPEC to bladder epithelial cells (94). However, these studies investigated the effect of P-pilus adhesion on bladder epithelial cells, whereas the Type 1 pilus is the essential adhesin for binding to the bladder (95–97). The bioavailability of cranberry constituents in the plasma and urine is well investigated in humans and provides the platform for comparison of products identified and the potential utility in humans (98–101). In addition, these studies have demonstrated that intact PACs are not excreted in human urine and thus are unavailable to prevent binding of bacteria to the urothelium (100, 101). Additional mechanistic studies also refute the activity of PACs in the urinary tract and suggest potential roles in the gastrointestinal tract (102–104). Our study did not reveal a single cranberry component that contributes to the phenotype. However, our observed potency with PURE juice and powder C that contained minimal levels of PACs is consistent with other studies that these molecules are likely not responsible for the effect of cranberry juice. Future studies will compare the components of medicinal foods that do not reduce filamentation to assist in defining the potential active molecules.

Historically, prophylactic use of cranberry juice has been discouraged due to the increased amount of sugar content consumed by the patient (82–86). In order to circumvent the added sugar, encapsulated cranberry powders have been manufactured, and the content has primarily been determined based on the levels of PACs. In addition, new formulations without any added sugar or sugar substitute are now currently available on the market, reducing the initial concerns of physicians. Some of the controversy regarding the use of cranberry products in the prevention and treatment of UTI may reflect the initial focus on PACs as the active ingredient, the lack of adequate characterization of some products, and the absence of mechanistic insight. In light of the accumulating data indicating that PACs may not be the most important cranberry constituent (102–104), it is important to reconsider the powder formulations provided in capsular form, or the use of unsweetened cranberry products. In summary, our observation that cranberry constituents prevent UPEC filamentation reveals a mechanism of action for cranberry derivatives in the treatment of UTI. Future studies will evaluate the efficacy of cranberry constituents in other pathogens known to use filamentation as part of the pathogenic lifestyle.

## SUPPLEMENTAL MATERIAL

Fig. S1 iai.00600-24-s0001.pdf Correlation of Flow cytometry events with viable bacterial counts.

ASM does not own the copyrights to Supplemental Material that may be linked to, or accessed through, an article. The authors have granted ASM a non-exclusive, world-wide license to publish the Supplemental Material files. Please contact the corresponding author directly for reuse.
