# Supplementary material for: Cranberry constituents prevent SOS-mediated filamentation of uropathogenic Escherichia coli
Source: Infect Immun. 2025 Apr 10;93(5):e00600-24. doi: 10.1128/iai.00600-24 (PMC12070744; doi:10.1128/iai.00600-24)
Supplement: Fig. S1 — Correlation of Flow cytometry events with viable bacterial counts. [file iai.00600-24-s0001.pdf]

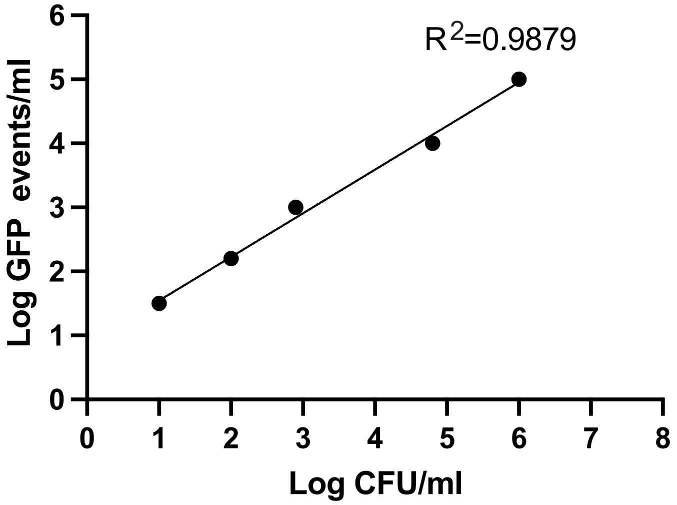

Supplemental Figure 1. **Correlation of Flow cytometry events with viable bacterial counts**.
Mid phase logarithmic (log) cultures of UTI89 pCOMGFP (optical density at 600 nm ~0.5) were serially diluted and analyzed in the logarithmic mode by flow cytometry using a combination of Forward scatter, Side Scatter and fluorescence to detect production of green fluorescent protein (GFP) as previously described (42). The same diluted cultures were plated on LB agar to enumerate viable bacteria. A linear correlation was observed between the log GFP positive cell count/ml and the log CFUs/ml over 6 orders of magnitude. The data points represent the average of 3 analyses performed on independent occasions. Linear correlation was determined in GraphPad Prism.
